# Dysregulation of prefrontal parvalbumin interneurons leads to adult aggression induced by social isolation stress during adolescence

**DOI:** 10.3389/fnmol.2022.1010152

**Published:** 2022-10-04

**Authors:** Xinyang Li, Huan Sun, Yuanyuan Zhu, Feidi Wang, Xiaodan Wang, Lin Han, Dongqi Cui, Danlei Luo, Yifang Zhai, Lixia Zhuo, Xiangzhao Xu, Jian Yang, Yan Li

**Affiliations:** ^1^Department of Anesthesiology and Perioperative Medicine & Center for Brain Science, The First Affiliated Hospital of Xi’an Jiaotong University, Xi’an, Shaanxi, China; ^2^Department of Neurobiology, Institute of Neurosciences, Fourth Military Medical University, Xi’an, Shaanxi, China; ^3^Key Laboratory of Environment and Genes Related to Diseases, Ministry of Education of China, Xi’an Jiaotong University, Xi’an, Shaanxi, China; ^4^Department of Diagnostic Radiology, The First Affiliated Hospital of Xi’an Jiaotong University, Xi’an, Shaanxi, China

**Keywords:** adolescent social isolation, aggression, social preference, PV interneurons, prefrontal infralimbic area

## Abstract

Social isolation during the juvenile stage results in structural and functional impairment of the brain and deviant adult aggression. However, the specific subregions and cell types that underpin this deviant behavior are still largely unknown. Here, we found that adolescent social isolation led to a shortened latency to attack onset and extended the average attack time, accompanied by anxiety-like behavior and deficits in social preference in adult mice. However, when exposed to social isolation during adulthood, the mice did not show these phenotypes. We also found that the structural plasticity of prefrontal pyramidal neurons, including the dendritic complexity and spine ratio, was impaired in mice exposed to adolescent social isolation. The parvalbumin (PV) interneurons in the prefrontal infralimbic cortex (IL) are highly vulnerable to juvenile social isolation and exhibit decreased cell numbers and reduced activation in adulthood. Moreover, chemogenetic inactivation of IL-PV interneurons can mimic juvenile social isolation-induced deviant aggression and social preference. Conversely, artificial activation of IL-PV interneurons significantly attenuated deviant aggression and rescued social preference during adulthood in mice exposed to adolescent social isolation. These findings implicate juvenile social isolation-induced damage to IL-PV interneurons in long-term aggressive behavior in adulthood.

## Introduction

Social isolation during COVID-19 may produce long-term negative cognition and behavior in humans. Social experiences during adolescence, a highly sensitive neurodevelopmental time window, are critical for establishing adult behaviors (Gogtay et al., 2004; Fuhrmann et al., 2015). Juvenile social isolation leads to inappropriate affection and behaviors, including anxiety (Loades et al., 2020), depression (Andersen and Teicher, 2008), drug abuse (Niño et al., 2016), and aggression (Davidson et al., 2000; Dodge et al., 2003; Wang et al., 2022), during adulthood in both humans and rodents.

The developmental maturity of the prefrontal cortex (PFC) is quite delayed compared with that of other brain regions, which is synchronized with the adolescent period (Rice and Barone, 2000; Arnsten, 2015). Increasing evidence supports that the prefrontal cortex is susceptible to various adolescent stresses, including social isolation (Tielbeek et al., 2018; Tan et al., 2021), electric foot shock (Lyttle et al., 2015), and high-fat food intake (Labouesse et al., 2017). However, the structural and functional alterations in specific prefrontal regions and cell types resulting from these adolescent stresses are still largely unknown. The PFC is a brain region in the frontal lobe and is anatomically separated into the orbital, lateral, and medial PFC (mPFC) (Dixon et al., 2017; Murray and Rudebeck, 2018). The rodent mPFC, comprising the anterior cingulate cortex (ACC), the prelimbic cortex (PrL), and the infralimbic cortex (IL), plays a critical role in modulating emotion, cognition, and behavior by integrating information through input and output projections (Rea et al., 2019; Chen et al., 2022).

The cell types in the mPFC are highly heterogeneous and include excitatory pyramidal neurons and various inhibitory interneurons (Tremblay et al., 2016). Neurons expressing parvalbumin (PV), somatostatin (SST), or vasoactive intestinal peptide (VIP) constitute the main functionally distinct inhibitory interneurons (Ascoli et al., 2008). Although the cell numbers of these inhibitory interneurons account for only 10-20% of the total neuronal population, they are crucial for the developmental maturity of the mPFC and local and distal neurotransmission (Hu et al., 2014; Kepecs and Fishell, 2014). In particular, prefrontal PV^+^ interneurons are highly plastic during adolescence, which causes these cells to be more vulnerable to environmental stress (Whittaker et al., 2011; Ruden et al., 2021). However, the role of juvenile prefrontal PV^+^ interneurons in adult aggressive behavior is still unclear.

Therefore, the present study aims to (1) determine the effect of adolescent social isolation on the cell number and activity of prefrontal PV^+^ interneurons in adults; (2) identify the role of prefrontal PV^+^ interneurons in aggressive behavior; and (3) investigate whether excessive aggression induced by adolescent social isolation can be rescued by modulating the activity of PV^+^ interneurons.

## Materials and methods

### Animals

Male C57Bl/6 wild-type mice and PV-Cre mice were used for the experiments. Male C57Bl/6 mice were obtained from Beijing Vital River Laboratory Animal Technology Co., Ltd. The PV-Cre mice were a gift from Dr. Hailong Dong (Department of Anesthesiology, Xijing Hospital) and were bred from one male and two females in an SPF environment. All mice were housed under standard laboratory conditions (temperature- and humidity-controlled conditions with a 12 h-12 h light-dark cycle with food and water available *ad libitum*). All animal experiments were approved by the Institutional Animal Care and Use Committees of Xi’an Jiaotong University (No. XJTULAC2020-1325). Animal-related procedures were performed in accordance with the National Institutes of Health guidelines, and every effort was made to minimize the suffering of animals and to reduce the number of animals used. A total of 242 mice aged 8–32 weeks were employed, of which 29 were eliminated owing to aggression screening, improper targeting, or inadequate viral vector expression.

### Social isolation

For adolescent social isolation (SI), male mice were randomly assigned to group-housed (GH) and SI conditions. Mice in the GH group were housed in groups of 4 mice per cage, and those in the SI group were individually housed in a standard mouse cage. On day 28 postnatal (P28), the protocol began and lasted until P63 (isolation for 5 weeks) (Makinodan et al., 2017; Deng et al., 2019). For the adult isolation condition, the protocol was the same as that of the adolescent group, with the exception that mice were isolated for 5 weeks between days 84 and 119.

### Virus injection and clozapine-N-oxide administration

Chemogenetic technologies were used to manipulate neurons in a cell type-specific fashion in freely moving animals. In order to inhibit PV^+^ interneurons, an mCherry tagged, adeno-associated virus (AAV) expressing hM4Di [Gi/o-coupled human muscarinic M4 designer receptor exclusively activated by a designer drug (DREADD)] was stereotaxically injected into PV-cre mice (Roth, 2016). With a similar principle to that of an inhibitory DREADD, the activating DREADD that is typically used to activate Gq signaling in neurons is hM3Dq. After viral expression, binding of the hM4Di or hM3Dq receptor to a specific ligand, clozapine N-oxide (CNO), can significantly reduce or enhance the firing of neurons, respectively.

PV-Cre mice were anesthetized with isoflurane (induction, 3%; maintenance, 1.5%) and placed in a rodent stereotaxic apparatus (RWD Life Science, Shenzhen, China). For mPFC-IL bilateral injections, the coordinates from bregma were –1.70 mm AP, ± 0.30 mm ML and –2.70 mm DV. Since recent studies argued that the CNO ligand may have endogenous effects in the mouse brain, we added a control virus group that expressed only mCherry to eliminate the interference of CNO (3 mg/kg) intraperitoneal injection. Each injection was infused at a rate of 50 nL/min using a microinjector and 10-μL Hamilton syringe containing 200 nL of AAV2/9-EF1α-DIO-hM4(DGi)-mCherry (5.45E + 12 vg/mL), AAV2/9-EF1α-DIO-hM3D(Gq)-mCherry (5.27E + 12 vg/mL) or AAV2/9-EF1α-DIO-mCherry (5.35E + 12 vg/mL). At the end of the infusion, the needle remained in place for 10 min to allow the virus to diffuse. All viruses used in this research were packaged by Brain VTA Co., Ltd., China. Behavioral experiments were conducted at least three weeks after the injection procedure to allow the mice to recover and the virus to be fully expressed. Moreover, the mice with low viral expression or an inaccurate injection site were excluded.

For behavior testing of inhibitory and excitatory DREADD manipulations to chemogenetically activate or inhibit IL-PV^+^ interneurons, CNO (stored at –20°C; C0832, Sigma) was dissolved in saline (0.9% NaCl solution) at a working concentration of 0.45 mg/ml and then administered intraperitoneally to hM3D- or hM4D-transfected mice (3 mg/kg body weight) 30 min prior to behavioral testing (Cheng et al., 2017; Burgdorf et al., 2020). Mice were designated to receive CNO and saline (SAL) in a counterbalanced fashion. To avoid potential confounding effects of CNO metabolites, CNO and SAL were administered to equal numbers of mCherry-transfected control animals. Each group of self-controlled experiments was separated by at least 2 days to ensure that the mice were well rested and that CNO metabolism was complete.

### Aggression screening

Mice that were injected with viruses and used in the chemogenetic experiments underwent aggression screening experiments one week prior to the resident-intruder (RI) experiments to ensure that the experimental mice had the potential for aggression (Flanigan et al., 2020). After acclimation to their home cage for at least 1 week, the experimental mice were exposed to a novel intruder (Kunming mice) for 10 min per day for 3 consecutive days. Each intruder presentation was performed between 14:00 and 17:00 h under white light conditions. The initial attack latency was recorded. Mice that showed aggressive behavior in one of the three experiments were considered to be aggressive and used in subsequent experiments. All aggression screening was halted if an intruder showed any signs of injury. The formal RI experiments and screening were separated by at least three days to ensure the normal state of the mice.

### Behavioral tests

#### Resident-intruder test

During RI sessions, subjects were faced with a smaller intruder (Kunming mice, body weight difference of 30%) for 10 min in their home cage. To keep the mice in their original habitat, we did not remove the lid of the cage while the experiment was in progress; instead, we videotaped the experiment by placing a camera outside the transparent cage wall. Aggressive behavior was evaluated by ethological analysis of video recordings, including the time from intruder entry to an initial attack and the total duration of aggression. The onset of aggression was defined as the first explicit physical antagonistic interaction initiated by the experimental mice (usually a bite), not including grooming or pursuit behavior. The attack was considered to have ended when the resident mouse reoriented itself away from the intruder after initiating the attack. Video analysis was performed manually frame by frame at a low speed, if necessary, by researchers who were blinded to the mouse groupings.

#### Open-field test

The open field experimental system is composed of a plug-in open field chamber (40 cm × 40 cm × 40 cm) and a data analysis and acquisition system (Panlab, SMARTPREMIUM). The bottom plate and four arms of the insert field are white, and the top is open. A digital camera was placed above the experimental box to record the activities of the mice. During the experiment, the mice were placed at the bottom of the box in the central position, and the activities of the mice inside the box were recorded over 10 min. The recording indexes mainly included the time and distance of the movement of each mouse in the central area and edge area. After each mouse test, the bottom and side walls of the box were cleaned with alcohol and dried with paper towels or rags to prevent any residual smell or feces from affecting subsequent experiments. The distance traveled reflects the spontaneous mobility of the mice, and the time spent in the central area reflects anxiety/depression; the shorter the central residence time is, the higher the anxiety level.

#### Free interaction test

Free interaction was used to evaluate social behaviors between two freely interacting mice in the open field apparatus. The mouse to be tested was first placed in the experimental chamber for 5 min to become familiar with the environment. Next, an unfamiliar male mouse (Kunming mice) was placed in the center of the open field. The test began the moment the intruder mouse was placed in the field, and the entire experiment lasted for 10 min. Both mice were allowed to move freely and socialize in the field. We recorded the behavior of the mice via the camera above. Videos were manually analyzed offline by an experimenter blind to the manipulations of the experimental mice and viewed frame by frame when necessary. Only attacks initiated by experimental mice toward intruder mice were recorded for the total duration of the attack. After each mouse was tested, the environment was cleaned with alcohol and wiped dry.

#### Three-chamber test

Testing was performed in the sociability cage, a three-chambered rectangular box (60 cm × 40 cm × 30 cm) with gray matte walls and floor, and the top was open. For habituation, the subject mouse was placed in the middle of the three-chamber apparatus to freely investigate for 10 min, with two empty cylindrical cages with transparent plastic fences located in the two opposite corners of the left and right chambers. Then, an age-, sex-, and strain-matched mouse was placed in the left or right chamber (systematically alternated) called the “social chamber,” and the subject mouse was allowed to move freely in the full chamber for 10 min of investigation. Behavior was tracked and scored by tracking software, including total exploration time, social cage investigation time and empty cage investigation time. The exploration time was defined as a nose point in the interaction zone, which is a circular zone with a radius of 2 cm surrounding the chamber containing the mouse or the empty chamber.

The social investigation preference index was calculated according to the following equation: social preference index = (social cage exploration time – empty cage exploration time)/(social cage exploration time + empty cage exploration time).

### Golgi-cox staining

The Golgi staining test was carried out according to previous reports with some modifications (Zhu et al., 2022). Mice were anesthetized with 5% pentobarbital and transcardially perfused with 30 ml of ice-cold PBS. The mouse brains were immersed in impregnation solution made by mixing equal volumes of commercial solutions. The Golgi-Cox solution, composed of 5% potassium dichromate, 5% mercuric chloride and a 5% solution of potassium chromate, was prepared in advance and protected from light for 1 week at room temperature. Subsequently, coronal sections were cut into 150 μm slices with a vibratome, and the slices were mounted on gelatin-coated slides and air-dried in the dark. The slides were rinsed in bidistilled water, stained with a mixture of solutions, and dehydrated in 50%, 75%, 95%, and 100% alcohol. Finally, the sections were cleared, mounted, and photographed with a confocal microscope (Olympus, Japan) and FLUOVIEW software (ver. 1.7a, Olympus, Japan).

The neuronal selection and tracing were carried out by a researcher who was blinded to the treatment assignments of the animals. Pyramidal neurons located in layers 2/3 and 5 of the PrL and IL regions of mice were selected. Before a pyramidal neuron was processed and morphologically analyzed, the following characteristics were confirmed: the neuron was uniformly labeled, was free of reactive precipitation, did not overlap with nearby cells, and was located in the middle of the section; the dendritic branches were generally parallel to the section; and the spines were visible (Nobili et al., 2017).

The dendritic branches and spines of neurons were analyzed using Imaris software (version 7.7.1, serial number: 32mr-rfhf-7hbu-jb58, Bitplane, Switzerland). The total length of dendrites and the volume of the cell body were automatically calculated. The selected dendrites included all apical and basal dendrites and primary and secondary dendrites. For the Sholl analysis, the spheres were constructed continuously from the center of the soma with an increasing radius of 10-μm increments. The number of intersections between each sphere and the dendrites was calculated for comparison. Sholl analysis using 3D images provides statistical results that are closer to the true structure of the neuron than using 2D images, especially when analyzing dendrites at different angles. The shape of the spines is defined by the length of the spine and the width of the spine neck and head, which allows us to classify spines into four types: stubby, mushroom, long thin and filopodia. The stubby type had a length < 1 mm; the mushroom type had a length > 3 mm and a maximum width of the head/average width of the neck > 2; the long thin type had a ratio of average width of the head/average width of the neck ≥ 1; and the remaining spines were filiform. Spine measurements were performed using the MATLAB-X Tension spine classifier in Imaris.

### Tissue preparation and immunofluorescent staining

To assess the activation of PV^+^ interneurons in the PrL and IL regions during natural aggression, mice were perfused with saline and 4% paraformaldehyde under deep anesthesia. The brains were fixed overnight at 4°C, and then the fixative was replaced with 30% sucrose solution for dehydration until the brains sank to the bottom of the container. The brains were cut into 30-μm-thick coronal sections with a freezing microtome (CryoStar NX50, Thermo, USA). Sections were washed in PBS three times (5 min each time) and permeabilized with PBS containing 0.5% Triton X-100 at 37°C for 30 min. Then, sections were blocked in 4% goat serum for 2 h at room temperature. The sections were incubated with the primary antibody at 4°C overnight (anti-parvalbumin, 1:500 concentration, Millipore, MAB1572, USA; c-Fos, 1:500 concentration, CST, #2250, USA). After three washes with PBS for 5 min each time, sections were incubated with species-specific fluorophore-conjugated secondary antibodies (Invitrogen A-11029 and A-21428) for 2 h at room temperature. Finally, DAPI was used to counterstain the nuclei after three washes in PBS. Images were acquired to verify virus expression and cell numbers. For c-Fos immunofluorescent staining, a total number of 16 C57Bl/6 mice (n = 4 from each group) were included.

### Statistical analyses

In experiments requiring viral infection of specific brain regions, mice were excluded from the behavioral analysis if they exhibited a significant change in motor responses as a result of the i.p. procedures or if the virus was found to be mislocalized or not expressed according to predetermined anatomical criteria. No data were excluded for other reasons. All data were tested for normality prior to analysis. For comparison of two experimental groups, two-tailed paired (Student’s, within-subject) *t* tests, an unpaired (between-subject) *t* test or two-way analysis of variance (ANOVA) followed by Tukey’s multiple comparisons was applied to data that conformed to a normal distribution; the two-tailed Wilcoxon matched-pairs signed rank test, the two-tailed Mann Whitney test or two-way ANOVA with Newman-Keuls multiple comparisons test was applied to data that was non-normally distributed. All tests were considered significant when *P* < 0.05. Statistical analysis was performed using GraphPad Prism 6 software.

## Results

### Adolescent social isolation stress increases aggression

**FIGURE 1:**
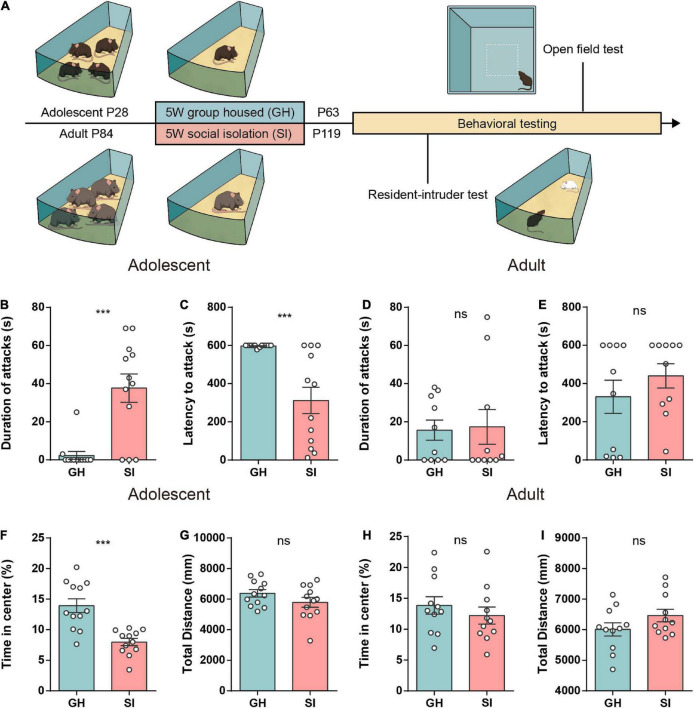
Mice exposed to chronic isolation stress during adolescence but not adulthood exhibit increased aggression and anxiety-like behavior. **(A)** Schematic of the social isolation model used in this study. **(B,C)** The duration **(B)** and latency **(C)** of aggression in the RI test of group-housed and socially isolated adolescent mice (*n* = 12 mice/group, duration of attack: GH, 2.33 ± 2.08, SI, 37.67 ± 7.44, ***P < 0.001; latency to attack: GH, 597.3 ± 1.94, SI, 311.8 ± 69.10, ***P < 0.001, *P* values were determined by the two-tailed Mann–Whitney test). **(D,E)** The duration **(D)** and latency **(E)** of aggression in the RI test of group-housed and socially isolated adult mice (*n* = 10 mice/group, duration of attack: GH, 15.63 ± 5.28, SI, 17.34 ± 9.12, *P* = 0.87 determined by a two-tailed unpaired *t* test; latency to attack: GH, 330.8 ± 87.13, SI, 440.1 ± 63.62, *P* = 0.41 determined by the two-tailed Mann–Whitney test). **(F,G)** In the open field test, socially isolated adolescent mice showed a significant decrease in time spent in the central area **(F)**. The total distance was similar between the two groups **(G)** (*n* = 12 mice/group; time in center, GH, 13.94 ± 1.13, SI, 7.98 ± 0.59, ***P < 0.001; total distance, GH, 6378 ± 239.0, SI, 5789 ± 321.2, *P* = 0.15 determined by a two-tailed unpaired *t* test). **(H,I)** The time spent in the center **(H)** and the total distance traveled **(I)** were similar between the two groups of adult mice (*n* = 11 mice/group; time in the center, GH, 13.84 ± 1.42, SI, 12.20 ± 1.38, *P* = 0.42; distance traveled, GH, 6011 ± 216.7, SI, 6464 ± 204.7, *P* = 0.14 determined by a two-tailed unpaired *t* test). Data are presented as the mean ± SEM. GH: group-housed; SI: social isolation.

To compare the behavioral effects of social isolation during adolescence and adulthood, we performed a 5-week single-housed intervention in both adolescent (4 weeks old) and adult mice (12 weeks old) to mimic chronic social isolation, which frequently promotes aggression in both humans and rodents (Wiberg and Grice, 1963; Zelikowsky et al., 2018; Tan et al., 2021). To eliminate the effect of the biological features of different age stages, we used age-matched group-housed mice as the control groups (Bicks et al., 2020) (Figure 1A). We used the RI test (Márquez et al., 2013; Wei et al., 2018) to measure aggression after 5 weeks of modeling. Behavioral tests revealed that adolescent SI mice exhibited a significantly longer attack duration (Figure 1B) and shorter latency to launch the first attack (Figure 1C) than adolescent group-housed (GH) mice. However, in adult mice, SI intervention did not significantly alter either attack duration (Figure 1D) or latency to initial aggression (Figure 1E) compared with the GH condition. In addition, to determine whether the heightened aggression was consistent, we then performed a free interaction experiment, a variant of the RI assay, to test the aggressive behavior in an open field. Notably, consistent with the RI test, 5 weeks of SI led to an increased attack duration in adolescent mice (Supplementary Figure 1) but not adult mice (Supplementary Figure 1).

Given that previous studies reported anxiety induced by long-term social isolation stress (Lin et al., 2015; Zelikowsky et al., 2018), we next performed an anxiety-like behavior test in both adolescent and adult mice. The adolescent SI mice, but not adult SI mice, spent less time in the center zone than their respective GH control mice (Figures 1F,H). Additionally, both adolescent and adult SI mice showed locomotor activity similar to that of the GH control mice (Figures 1G,I). Moreover, we found that although both adolescent SI and adolescent GH mice showed a higher preference for the social cage than the empty cage in the three-chamber test, the adolescent SI mice showed a significant reduction in the exploration time of the social cage (Supplementary Figure 1) and social preference index (Supplementary Figure 1) compared with GH mice.

Collectively, these findings demonstrate that mice exposed to chronic social deprivation during adolescence, a sensitive developmental period, show not only excessive aggression but also increased anxitey-like behaviors and social preference deficits compared with mice exposed to the same isolation stress during adulthood.

### Mice subjected to chronic adolescent social isolation stress show aberrant dendritic structures and spine ratios in medial prefrontal cortex pyramidal neurons

According to previous studies, critical maturation of the PFC region continues throughout adolescence in both humans and rodents (Miller et al., 2019). During maturation, the number of dendritic spines successively increase until reaching a peak and then it declines to adult values by appropriate pruning during adolescence (Elston et al., 2009).

To better understand whether and what changes occurred in the mPFC, including the PrL and IL regions, during the sensitive time window of adolescence, we examined the structural plasticity of the synapses in SI mice.

**FIGURE 2:**
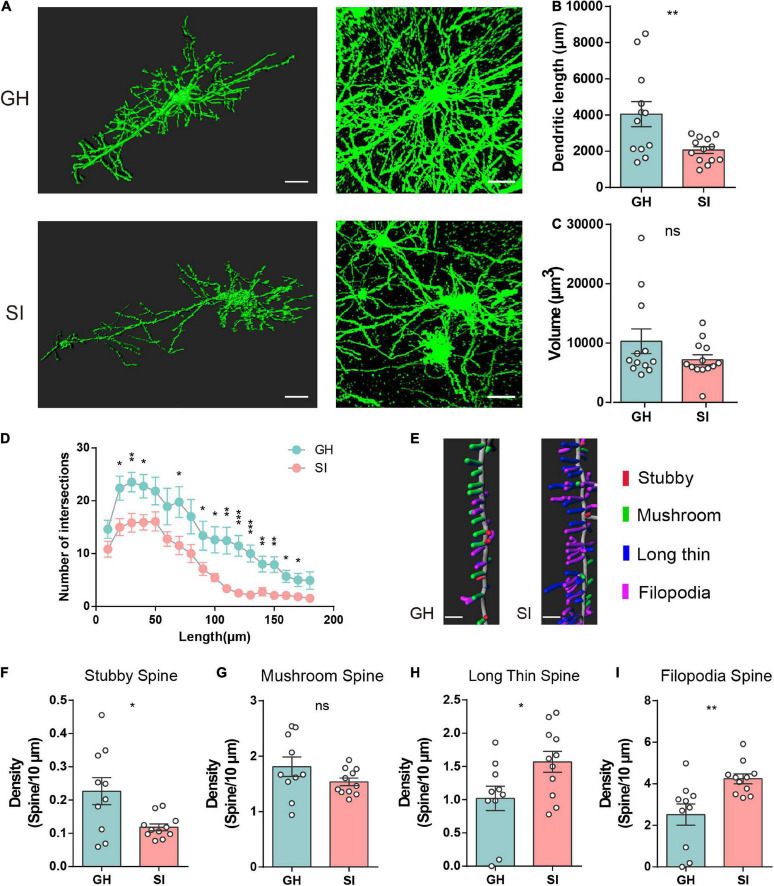
Impacts of adolescent social isolation on the structure of dendrites and spines of pyramidal neurons in the mPFC. **(A)** Representative reconstructed (left) and raw (right) images of pyramidal neurons in the PL and IL regions of GH- and SI-treated mice. Scale bars, 30 μm, left; 50 μm, right. In the adolescent-GH group, 4 neurons in layer 2/3 and 8 neurons in layer 5 from 3 mice were used; in the adolescent-SI group, 5 neurons in layer 2/3 and 8 neurons in layer 5 from 3 mice were used. **(B,C)** Summary of dendritic length **(B)** and volume **(C)** of PL and IL pyramidal neurons obtained from the experimental mice (GH, *n* = 12; SI, *n* = 13, dendrite length, GH, 4055 ± 690.9, SI, 2067 ± 188.4, two-tailed unpaired *t* test: *P* < 0.01; volume, GH, 10309 ± 2068, SI, 7196 ± 850.3, two-tailed Mann–Whitney test *P* = 0.40). **(D)** Sholl analysis of dendritic branching complexity in the basal and apical dendrites of the two groups of mice. (GH, *n* = 13; SI, *n* = 13; *P < 0.05, **P < 0.01 and ***P < 0.001 determined by two-tailed unpaired *t* test or Mann–Whitney test). **(E)** Representative confocal 3D reconstruction images of dendritic spines of PL or IL pyramidal neurons obtained from GH and SI mice. Scale bars, 5 μm. **(F–I)** Summary of the density of stubby- **(F)**, mushroom- **(G)**, long thin- **(H)**, and filopodia- **(I)** shaped spines on the dendrites of pyramidal neurons in the PL and IL regions of GH and SI mice. (GH, *n* = 10, SI, *n* = 11, two-tailed unpaired *t* test: stubby spines, GH, 0.23 ± 0.04, SI, 0.12 ± 0.01, *P* < 0.05; mushroom spines, GH, 1.81 ± 0.18, SI, 1.54 ± 0.07, *P* = 0.15; long thin spines, GH, 1.02 ± 0.18, SI, 1.57 ± 0.16, *P* < 0.05; filopodia spines, GH, 2.52 ± 0.51, SI, 4.24 ± 0.23, *P* < 0.01). Data are presented as the mean ± SEM. GH: group-housed; SI: social isolation.

The dendritic length in the adolescent SI group was shorter than that in the corresponding GH group (Figures 2A,B), and no significant changes were observed between adult SI and GH mice (Supplementary Figure 2B). However, we did not find obvious changes in the total volume of dendrites in adolescent or adult SI mice (Figure 2C and Supplementary Figure 2C). Furthermore, the dendritic complexity of pyramidal neurons was significantly different between adolescent SI mice and GH mice, and the number of dendritic intersections was reduced in the mPFC of adolescent SI mice (Figure 2D); however, no differences between adult SI and GH mice were observed within the 110 μm radius (Supplementary Figure 2). These findings indicated that the dendritic complexity was impaired during adolescent social isolation.

We next measured the ratios of synaptic spines with high-resolution analysis, and all spines were classified into four categories on the basis of their morphology: stubby-, mushroom-, long/thin-, and filopodia-shaped subtypes (Zagrebelsky et al., 2005; Bian et al., 2015). Stubby- and mushroom-shaped spines are generally identified as mature spines, but long thin and filopodia spines are considered more plastic and immature (Tada and Sheng, 2006; Bourne and Harris, 2007). Here, we found that adolescent SI is associated with a selective reduction in stubby-shaped spines (Figures 2E,F) but does not affect the density of mushroom-like spines (Figure 2G). Moreover, the numbers of both long thin- (Figure 2H) and filopodia-shaped (Figure 2I) spines were prominently increased in the mPFC of adolescent SI mice compared with GH mice. However, there were no significant differences in the densities of these four types of spines between adult SI and GH mice (Supplementary Figures 2F–I). Together, these results suggest an important long-range influence on the morphology and synaptic plasticity of pyramidal neurons in the mPFC of mice exposed to chronic social isolation during adolescence but not during adulthood.

### Chronic social isolation during adolescence leads to decreases in the quantity and activity of IL-PV^+^ interneurons

There is accumulating evidence that PV^+^ interneurons play a critical role as the period trigger in relation to plasticity onset in the cortex (Markram et al., 2004; Hensch, 2005). Given the combined results of previous studies showing that chronic stress may downregulate PV expression (Jiang et al., 2013), we wondered whether chronic social isolation stress-induced synaptic structural deficits are accompanied by impairment of PV^+^ interneurons.

**FIGURE 3:**
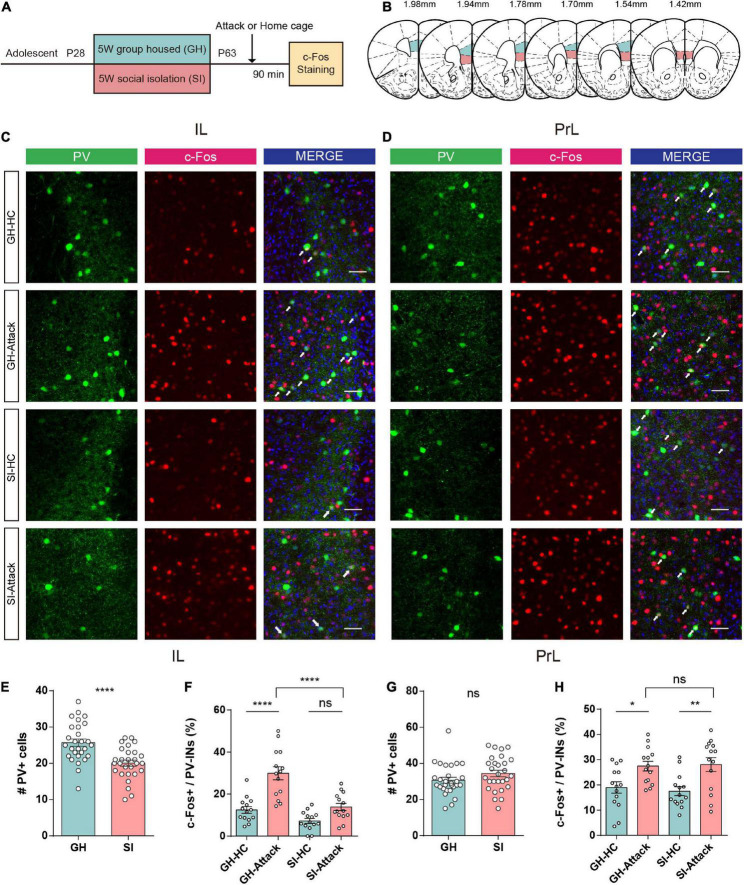
Adolescent social isolation induces a decrease in the quantity and activity of PV^+^ interneurons in the IL subregion. **(A)** Schematic flow chart for c-Fos immunofluorescent staining. **(B)** The six sequential brain atlas slides from bregma levels 1.98 mm to 1.42 mm delineating the target PFC subfields of interest. Green, PrL; pink, IL. **(C,D)** Confocal images showing the expression of PV^+^ interneurons (green), c-Fos (red), and the merged image in the IL **(C)** and PrL **(D)** subregions. The brain slices obtained from GH and SI mice in the home cage or after active attacks (white arrows indicate cells colabeled for PV, c-Fos and DAPI. Scale bar, 50 μm). **(E)** The number of PV^+^ interneurons in the IL decreased after 5 weeks of social isolation during the adolescent period (*n* = 28 slices/8 mice/group, GH, 25.71 ± 1.01, SI, 20.04 ± 0.84, *P* < 0.0001 determined by two-tailed unpaired *t* test). **(F)** GH mice show increased numbers of c-Fos-positive PV^+^ interneurons after aggression. SI mice showed no difference in the number of activated PV^+^ interneurons in the IL (*n* = 14 slice/4 mice/group, GH-HC, 12.46 ± 1.57; GH-Attack, 29.90 ± 3.10; SI-HC, 7.19 ± 1.18; SI-Attack, 13.84 ± 1.72, ****P < 0.0001 determined by two-way ANOVA with Tukey’s multiple comparison test). **(G)** No changes occurred in the number of PV^+^ interneurons in the PrL after 5 weeks of social isolation during adolescence (*n* = 28 slices/8 mice/group, GH, 30.68 ± 1.64, SI, 34.43 ± 1.79, *P* = 0.13 determined by two-tailed unpaired *t* test). **(H)** The number of c-Fos-positive PV^+^ interneurons increased after aggression compared to the home cage condition in the PrL of both GH and SI mice, and there was no obvious difference between the GH-attack and SI-attack groups (n = 14 slices/4 mice/group, GH-HC, 18.94 ± 2.27; GH-Attack, 27.46 ± 1.91; SI-HC, 17.49 ± 1.77; SI-Attack, 28.06 ± 2.74, *P < 0.05, **P < 0.01 determined by two-way ANOVA with Tukey’s multiple comparison test). All data in **(E–H)** are presented as the mean ± SEM. GH: group-housed; SI: social isolation.

We first performed immunofluorescent costaining for PV^+^ interneurons and the immediate early gene c-Fos in tissues from adolescent SI and GH mice (Figure 3A) 1.5 h after aggression. The regions of interest are shown in Figure 3B for PrL and IL. The numbers of PV^+^ interneurons in the IL (Figures 3C, left and E) were significantly reduced under the SI condition compared with the GH condition. However, the number of PV^+^ interneurons in the PrL was similar between the SI and GH groups (Figures 3D, left and G).

The number of c-Fos-labeled PV^+^ cells in the IL was conspicuously increased in GH mice after attack compared with home cage condition. However, the proportion of c-Fos-labeled PV^+^ cells in the IL was not significantly changed after attack in SI mice. In addition, the proportion of activated PV^+^ interneurons in the IL of SI mice was significantly smaller than that of GH mice (Figures 3C,F), suggesting a failure of PV^+^ interneuron activation upon aggressive encounters in SI mice.

In contrast, we did not observe a similar discrepancy in activation of PV^+^ interneurons in the PrL subregion after attack between SI and GH mice. In the PrL, PV^+^ interneurons exhibited elevated c-Fos expression in both SI and GH mice after aggression compared with the home cage condition (Figures 3D,H). Collectively, these data indicate that adolescent social isolation profoundly impacts the numbers and activation of PV^+^ interneurons in the IL subregion of the PFC but not in the PrL subregion, which may underlie SI-induced aggression.

### Inhibition of IL-PV^+^ interneurons increased aggression in group-housed mice

**FIGURE 4:**
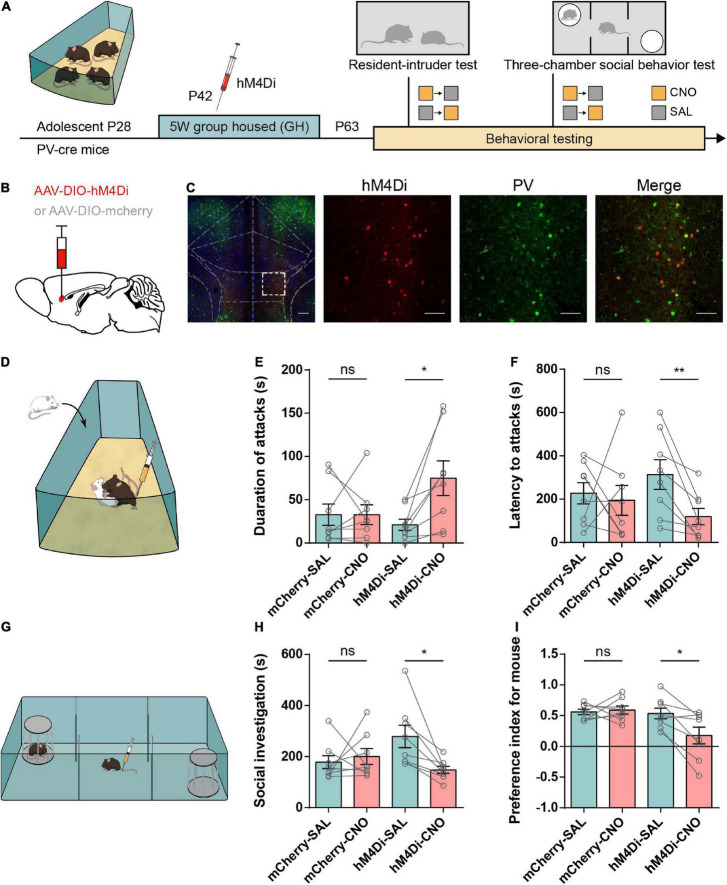
Chemogenetic inhibition of IL-PV interneurons increases aggressive behaviors and disrupts social preference in group-housed mice. **(A)** Timeline showing injection of the Cre-dependent inhibitory virus hM4Di or mCherry into the IL of group-housed PV-Cre mice and the subsequent repeated behavioral tests. Every mouse was tested under both CNO (yellow square) and SAL (gray square) conditions, and each behavioral test was counterbalanced by the order of the injections. **(B)** Strategy of specifically expressing inhibitory G-protein (Gi)-coupled hM4Di receptor or mCherry in PV^+^ interneurons. **(C)** Representative images of inhibitory hM4Di preferentially injected into IL-PV^+^ interneurons (left, scale bar, 200 μm) and colocalization of hM4Di virus expression (red) with PV staining (green) (right, scale bar, 100 μm). **(D)** Experimental scheme for inhibiting hM4Di expression in IL-PV^+^ interneurons 30 min before the resident-intruder test. **(E)** In the GH group, CNO treatment of hM4Di mice but not SAL promoted increased aggression, and mCherry mice showed no substantial changes under either the SAL or CNO condition (*n* = 8 mice/group, mCherry, SAL, 32.81 ± 12.29, CNO, 32.75 ± 11.55, *P* = 0.94 obtained by two-tailed Wilcoxon matched-pairs signed rank test; hM4Di, SAL, 21.13 ± 6.52, CNO, 74.94 ± 19.99, *P* < 0.05 obtained by two-tailed paired *t* tests). **(F)** Only GH mice expressing hM4Di under CNO conditions showed a reduced latency to attack, and mCherry mice or hM4Di mice injected with SAL showed no substantial changes (*n* = 8 mice/group, two-tailed paired *t* test: mCherry, SAL, 227.4 ± 49.25, CNO, 194.9 ± 68.89, *P* = 0.74; hM4Di, SAL, 313.9 ± 68.13, CNO, 119.7 ± 37.65, *P* < 0.01). **(G)** Mice were injected with either 3 mg/kg CNO or SAL 30 min before the 3-chamber social behavior tests. **(H)** Bar graphs showing that inactivation of the IL-PV^+^ interneurons decreased the time spent in social investigation (*n* = 8 mice/group, mCherry, SAL, 178.3 ± 25.60, CNO, 200.4 ± 30.93, *P* = 0.64 obtained by two-tailed Wilcoxon matched-pairs signed rank test; hM4Di, SAL, 279.0 ± 44.14, CNO, 147.9 ± 13.84, *P* < 0.05 obtained by two-tailed paired *t* tests). **(I)** Inactivation of IL-PV^+^ interneurons decreased the social preference index of GH mice (*n* = 8 mice/group, two-tailed paired *t* tests: mCherry, SAL, 0.56 ± 0.04, CNO, 0.59 ± 0.06, *P* = 0.74; hM4Di, SAL, 0.53 ± 0.09, CNO, 0.18 ± 0.14, *P* < 0.05).

Based on the hypofunction of IL-PV^+^ interneurons in adolescent SI mice, we next asked whether inhibiting these PV^+^ interneurons in GH mice would induce a similar increase in attack behavior. The Cre-dependent adeno-associated virus (AAV2) was bilaterally injected into the IL of PV-Cre mice to express the engineered Gi-coupled hM4D receptor (Armbruster et al., 2007) along with the fluorescent reporter mCherry. Figures 4B,C depict the viral injection site and the spread of virus indicated by the presence of mCherry. The virus was primarily limited to the IL region and highly specific to PV^+^ interneurons.

We then tested the influence of IL-PV^+^ interneuron suppression in GH mice by using a battery of behavioral tests, including the RI test for aggression, 3-chamber test for social preference, and an open field (Figure 4A). Mice were intraperitoneally injected with CNO or saline (Labouesse et al., 2017) 30 min prior to testing (Figure 4D). Suppression of IL-PV^+^ interneurons induced a significant elevation in the attack duration and a corresponding decrease in the latency to the initial attack, while we did not detect altered aggression in mCherry-injected mice treated with CNO or SAL (Figures 4E,F). The above results represent the increased aggression induced by functional lesions of IL-PV^+^ interneurons.

In the 3-chamber test (Figure 4G), the time spent in social investigation showed a decreasing trend only in hM4Di-expressing mice with CNO injection compared to those that received SAL injection (Figure 4H). Furthermore, treatment with CNO also led to a pronounced reduction in the preference index for mice in the hM4Di group but not in the mCherry group (Figure 4I), suggesting a decrease in social preference.

In addition, neither hM4Di- nor mCherry-expressing mice showed obvious alterations in time spent in the center and total distance traveled after CNO treatment in the open field test, indicating unchanged anxiety and locomotion (Supplementary Figures 3A–D). Taken together, these findings demonstrate that suppression of PV^+^ interneurons in the IL not only causes excessive aggression but also disrupts social preference without impacting anxiety or locomotor activity, suggesting that adequate IL-PV^+^ interneuron activity is physiologically necessary for normal social behaviors.

### Activation of IL-PV^+^ interneurons alleviates adolescent social isolation-induced aggression

**FIGURE 5:**
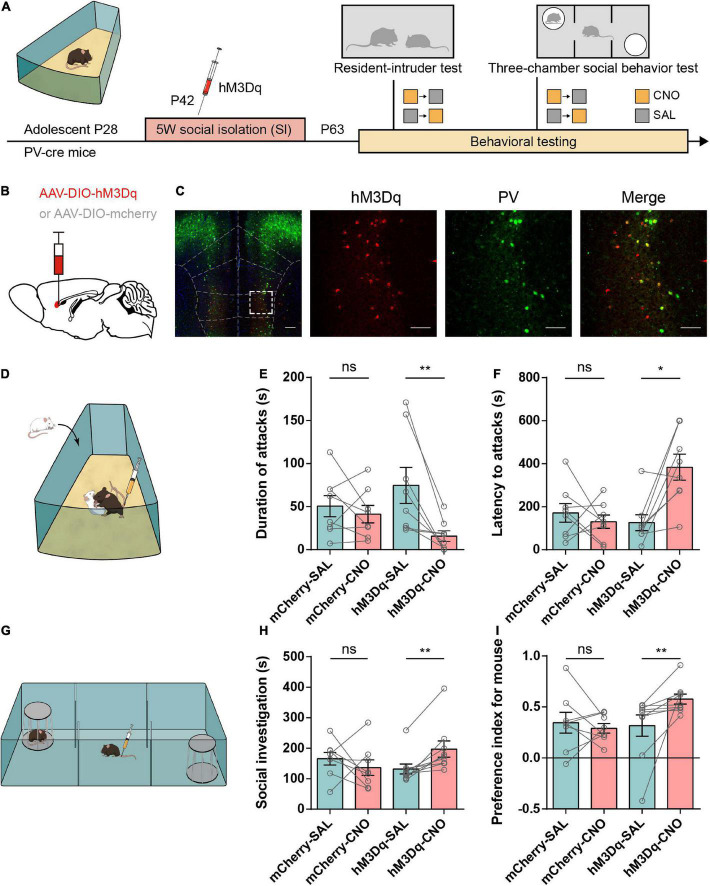
Activation of IL-PV^+^ interneurons attenuates aggressive behaviors and rescues impaired sociability in adolescent social isolation mice. **(A)** Experimental design showing adolescent social isolation and a schematic of the injection of the Cre-dependent activation virus hM3Dq into SI PV-Cre mice. Aggression and sociability behavior testing were carried out three weeks later. **(B)** Strategy of specifically expressing the excitatory G-protein (Gq)-coupled hM3Dq receptor or mCherry in PV^+^ interneurons. **(C)** Overlay of hM3Dq or mCherry expression in IL PV^+^ interneurons. Immunofluorescent images of a brain slice showing the example location of hM3D (Gq) expression in the IL region (left, scale bar, 200 μm) and colocalization between hM3Dq (red) and PV staining (green) (right, scale bar, 100 μm). **(D)** Experimental scheme for hM3Dq expression in the resident-intruder test. **(E,F)** Behavioral results from the resident-intruder test for aggressivity show that only the use of CNO in hM3Dq-expressing SI mice decreases the duration of attacks **(E)** (*n* = 8 mice/group, two-tailed paired *t* test: mCherry, SAL, 50.50 ± 12.20, CNO, 41.25 ± 10.08, *P* = 0.48; hM3Dq, SAL, 74.69 ± 20.88, CNO, 15.81 ± 6.173, *P* < 0.01) and increases the latency to attack **(F)** (*n* = 8 mice/group, mCherry, SAL, 171.6 ± 43.55, CNO, 130.6 ± 31.09, *P* = 0.51 determined by two-tailed paired *t* test; hM3Dq, SAL, 126.1 ± 37.17, CNO, 383.9 ± 60.42, *P* < 0.05 obtained by two-tailed Wilcoxon matched-pairs signed rank test). **(G)** Experimental scheme for hM3Dq activation of IL-PV^+^ interneurons before the 3-chamber test. **(H,I)** In SI mice, the 3-chamber test for sociability showed that mice treated with hM3Dq showed increases in time spent in social investigation **(H)** (mCherry group, *n* = 8 mice, SAL, 165.6 ± 20.83, CNO, 136.4 ± 25.33, *P* = 0.44 determined by two-tailed paired *t* test; hM3Dq group, *n* = 9 mice, SAL, 131.8 ± 16.38, CNO, 197.2 ± 26.74, *P* < 0.01 determined by two-tailed Wilcoxon matched-pairs signed rank test) and social preference **(I)** (mCherry group, *n* = 8 mice, SAL, 0.35 ± 0.10, CNO, 0.29 ± 0.05, *P* = 0.60 determined by two-tailed paired *t* test; hM3Dq group, *n* = 9 mice, SAL, 0.32 ± 0.11, CNO, 0.58 ± 0.05, *P* < 0.01 determined by two-tailed Wilcoxon matched-pairs signed rank test) only after CNO administration.

We wanted to further determine whether reactivation of IL-PV^+^ interneurons would be sufficient to correct the excessive aggression induced by adolescent SI. IL-PV^+^ interneurons were chemogenetically stimulated by bilateral injection of an excitatory designer receptor exclusively activated by the Gq-coupled hM3D receptor in the SI-exposed PV-Cre mice, while the control group received only a control virus containing a fluorescent mCherry protein. SI PV-Cre mice expressing hM3D were tested in a variety of behavioral tests, including the RI, 3-chamber and open field tests (Figure 5A). Then, we confirmed the targeting of the injection site using immunofluorescence costaining to validate the specificity of the virus in IL-PV^+^ interneurons (Figures 5B,C).

Resident-intruder (RI) tests were performed 3 weeks after the operations, and all mice received CNO or SAL injections 30 min before the test (Figure 5D). We observed a markedly reduced attack duration and prolonged latency to the first attack onset in the hM3D group after CNO injection compared to SAL injection (Figures 5E,F). However, the aggressive behaviors in the mCherry group after CNO or SAL injection were similar. These data suggest that IL-PV^+^ interneuron activation contributes to mitigating excessive aggression caused by adolescent social isolation.

In parallel, social investigation, reflected by the social preference index, was significantly rescued in hM3D-CNO-treated PV-Cre mice compared with hM3D-SAL-treated mice. There was no obvious change in sociability between mCherry-CNO-treated and mCherry-SAL-treated mice. Altogether, these data indicate that activation of IL-PV^+^ interneurons is capable of alleviating social preference deficits in adolescent SI mice (Figures 5G–I).

Moreover, we found that activation of IL-PV^+^ interneurons markedly increased the center time of adolescent SI mice (Supplementary Figures 3E,F) without affecting the general locomotor activity (Supplementary Figure 3G). Therefore, adolescent SI-induced anxiety-like behavior can be alleviated by activation of IL-PV^+^ interneurons. These results revealed that stimulation of IL-PV^+^ interneurons during adulthood is sufficient to ameliorate excessive aggression and anxiety and rescue social preference in mice exposed to adolescent SI.

## Discussion

### Summary

**FIGURE 6:**
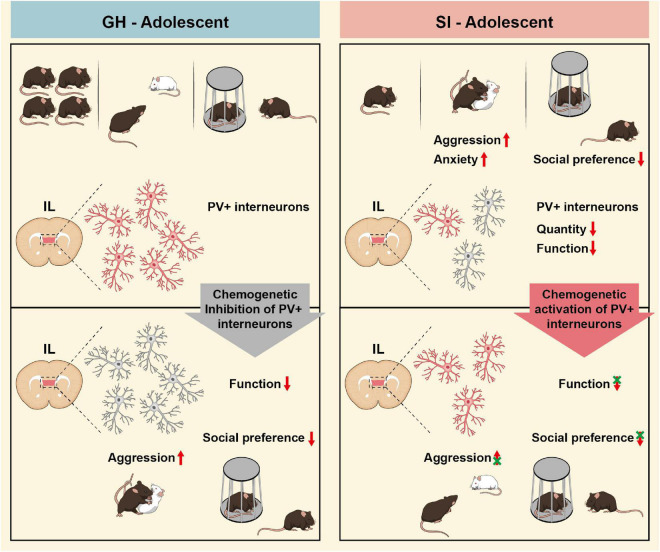
A Summary scheme shows the proposed role of IL-PV^+^ interneurons in adolescent social isolation-induced aggression. **(Left panel)** Adolescent GH mice exhibit typical development, including normal social behaviors and healthy IL-PV^+^ interneurons. Increased aggression and decreased social preference can be induced in GH mice by inhibiting IL-PV^+^ interneurons. Right panel, adolescent SI leads to excessive aggression, impaired social preference and increased anxiety compared to group housing. IL-PV^+^ interneurons show decreases in quantity and intrinsic excitability in adolescent social isolation mice. Conversely, the excessive aggression, impaired sociability, and anxiety-like behaviors can be alleviated by activation of IL-PV^+^ interneurons in these adolescent SI mice. GH: group-housed; SI: social isolation.

In this study, we investigated the relationship between adolescent social isolation-induced brain alterations and aggressive behavior. We demonstrate that after adolescent social isolation, the developmental processes in the mPFC brain region of mice were significantly impaired, not only by reduced synaptic plasticity of pyramidal neurons but also by a decrease in the quantity and activity of PV^+^ interneurons in the IL region, which was accompanied by excessive aggression, increased anxiety-like behavior and diminished social preference. Chemogenetic inhibition of IL-PV^+^ interneurons can mimic the abnormal aggression and social preference deficits in mice exposed to adolescent social isolation. Conversely, activation of PV^+^ interneurons prevented SI mice from exhibiting increased aggression and impaired social preference (Figure 6). Our study promotes an understanding of IL-PV^+^ interneuron involvement in adolescent social isolation, which provides the ability to directionally regulate aggression and social preference behaviors.

### Adolescence is a special sensitive period for neural development

Adolescence is a unique developmental window during which the individual undergoes rapid development up to maturity and is therefore generally considered to be susceptible to many diseases. During this time, chronic stresses impact the developmental process, and some effects last even to adulthood. As in prior studies, long-term social deprivation in early life can impair sociability (Fone and Porkess, 2008; Bicks et al., 2020; Lee et al., 2021); thus, the complex peer-peer social interaction during the adolescent period is considered to be an important part of the development of experience-dependent plasticity that persists into adulthood (Walker et al., 2019).

To date, it is unknown whether similar exposure to chronic social isolation stress during adolescence has a persistent effect on adults. This study investigated the effects of adolescent social isolation on aggression, social preference and anxiety in mice and, for the first time, compared the different effects of adolescent social isolation in mice with those of adult social isolation. Our results show that following the same 5-week social isolation period during adolescence but not during adulthood, mice exhibit heightened aggression, increased anxiety-like behavior, impaired social preference, and morphological changes in pyramidal neuronal plasticity. Our findings shed light on a second sensitive window of development and are in line with age differences in stress tolerance in humans regarding stress-related mental disorders. Moreover, they can be applied to explain why children exposed to social isolation during adolescence may exhibit abnormal behavior in adulthood, which also suggests the importance of appropriate developmental conditions for adolescence.

### Adolescence social isolation leads to abnormal maturation of medial prefrontal cortex neurons

Adolescence constitutes an important period for the refinement of glutamatergic and GABAergic function in the PFC, compared with other brain regions, in both primates and rodents (van Duijvenvoorde et al., 2016), and the developmental process lasts until adulthood (Uhlhaas et al., 2010; Caballero et al., 2021).

At the level of dendritic spines, among the four types of dendritic spines, large spines, such as stubby and mushroom spines, form stronger, longer-lasting synapses, while the long thin and filopodia spines are generally transient, forming weaker synapses (Kasai et al., 2003; Sebastian et al., 2013). An increase in the number of long-thin spines with a concomitant decrease in that of stubby spines identifies a shift in spine stability as a consequence of social defeat stress (Iñiguez et al., 2016). According to our results, mice that experienced adolescent social isolation showed a decrease in the abundance of mature stubby spines; thus, their functional activity may have been affected. The number of relatively weak long thin and filopodia spines increased significantly, and this substantial increase may impact the functional activity of mature dendritic spines and also indicate an impact on pruning during development.

During this strict time window, the opening of the critical period is triggered by the maturation of PV^+^ interneurons that are centrally involved in inhibitory functions (Donato et al., 2013; Toyoizumi et al., 2013). The subsequent development is followed by an optimal reduction in the excitatory/inhibitory (E/I) balance, as an abnormally disinhibited PFC has been linked to functional impairment and fails to attain the hallmark of adult-like function (Gruber et al., 2010).

Thus, we inferred that there may be functional and developmental faults in PV^+^ interneurons, which are likely to be the basis of the excessive aggression. By counting PV^+^ interneurons, as well as detecting their activation during aggression, our results confirm that specifically in the IL subregion, PV^+^ interneurons show significant reductions in number and functional impairments during this chronic stress.

It has been previously shown that after long-term chronic stress, rats show a significant reduction in the number of PV^+^ interneurons in the mPFC, especially in the IL brain region (Czéh et al., 2018). Although the paradigms and durations were different from those in our study, as shown in our immunofluorescence results, neuronal degeneration, death, or the loss of signature protein expression of the PV^+^ interneurons affected the labeling of these cells in the immunofluorescence staining, indicating a substantial functional decline of PV^+^ interneurons in response to this kind of stress intervention, which may lead to an imbalance in the E/I ratio of the IL and consequently to the loss of proper regulation in aggressive behavior.

However, the dendritic changes in PV^+^ interneurons as well as the mechanisms by which PV^+^ interneurons affect mouse behavior by altering the plasticity of pyramidal neurons still require further investigation.

### The multifaceted role of the infralimbic cortex subregion

We focused our attention on the IL subregion, as it is the most sensitive subregion of the PFC to chronic stress, supported by our comparison of PV^+^ interneuron activity during aggression in both adolescent SI and GH mice. IL has been shown to be involved in a wide range of behaviors, including fear extinction (Phelps et al., 2004), empathy (Shamay-Tsoory et al., 2009), anxiety (Sullivan and Gratton, 2002), social preference (Huang et al., 2020), social recognition (Park et al., 2021) and aggression (Biro et al., 2018). Moreover, as the IL plays a role in value judgments, decision making and empathy, people with IL impairment exhibit a lack of remorse (Camille et al., 2004; Krajbich et al., 2009), as well as aberrations in moral judgment (Fumagalli and Priori, 2012). Together, the regulatory effects of the IL on attack, the evidence supporting the negative effects of IL damage and our findings validate the modulation of abnormal aggression by the IL. In addition, the regulation of sociability by mPFC-PV^+^ interneurons found in prior studies (Bicks et al., 2020) can corroborate the positive and negative impacts on social preference when we modify IL-PV^+^ interneurons. Moreover, the distinct IL outputs showed completely opposite effects of anxiety regulation, as the IL to the lateral septum (LS) projections promote anxiety whereas the IL to the central nucleus of the amygdala (CeA) projections exert anxiolytic activity (Chen et al., 2021); this may also explain our findings that activation of IL-PV^+^ interneurons is able to rescue anxiety, but inhibition of the IL is not sufficient to increase anxiety, suggesting that other mechanisms may still have a synchronous effect in the control of anxiety-like behaviors.

### Modulation of aggressive behaviors by the medial prefrontal cortex region

Aggression is a highly conserved behavior throughout evolution. By means of fighting, social hierarchies set up, and valuable resources such as mates, food, and territory are protected. Thus, aggressiveness *per se* has never been considered abnormal. However, excessive aggression is considered pathological behavior that is linked to many psychiatric disorders, most notably borderline personality disorder (Chu et al., 2022), antisocial personality disorder, and intermittent explosive disorder (Siever et al., 1999). For humans, violent behavior, especially among youth, has aroused great concern due to the unpredictable threat to the lives of individuals and adverse effects on social security. Hence, the modulation of aggressive behavior by various brain regions and distinct neural circuits has been a focus of attention. Classic works concluded that instinctive behaviors, including aggression, are triggered by sensory input and mediated by circuits of motor output and that the core aggression circuit includes the medial amygdala (Hong et al., 2014), the bed nucleus of the stria terminalis (Padilla et al., 2016), ventromedial hypothalamus (Lee et al., 2014; Lin et al., 2015) and ventral premammillary nucleus (Motta et al., 2013). In fact, after the classic case in which a patient with PFC damage showed a higher propensity to attack (Anderson et al., 1999) was reported, the regulatory effect of the PFC on aggression gradually began to attract attention. Furthermore, a seminal aggression theory stating that weakness in “top-down” control systems in turn leads to increased aggressive behaviors confirmed the important inhibitory effect of the PFC on the limbic system in regulating aggression (Larry and Siever, 2008). Nevertheless, top-down modulation in response to attacks remains poorly understood, and existing studies have mainly focused on PFC pyramidal neurons and the associated outward projections (Tan et al., 2021; Wang et al., 2022). The current study is the first to suggest the mechanisms of advanced cortical regulation of aggression at the microcircuit level and reveal an important role of PV^+^ interneurons in the regulation of aggression in specific IL subregions.

### Crucial yet vulnerable PV^+^ interneurons

As they are critically involved in microcircuits and neuronal networks (Hu et al., 2014), PV^+^ interneurons are considered major regulators of the E/I balance in cortical circuits (Le Magueresse and Monyer, 2013). Furthermore, due to their extensive targeting and unique morphological and electrophysiological features, PV neurons can connect with a large number of cells (Hu et al., 2014). Thus, inhibition of a limited number of PV neurons has the potential to have an extensive impact on network activity. Lines of evidence have shown that PV^+^ neurons are vulnerable to stress, and chronic stress not only decreases the number of PV-immunoreactive neurons (Czeh et al., 2005) but also impairs the generation of rhythmic spontaneous inhibitory postsynaptic currents (IPSCs), especially those originating from PV^+^ interneurons (Hu et al., 2010). Moreover, simply downregulating PV neurons to the adolescent level can lead to a dysregulation of E/I balance that reduces the ability of PV neurons to integrate afferent information and ultimately impairs the optimal computational capacity of the PFC (Caballero et al., 2020). Therefore, based on the existing findings, we suggest that there are two possible patterns of PV neurons that regulate aggressive behavior.

### Potential mechanisms by which PV^+^ interneurons regulate aggression

One involves modulating the projections of its targeted pyramidal neurons to downstream brain regions. Although long-term PFC damage can result in an increased propensity to aggression, the execution of attack still requires heightened PFC activity. Existing studies have shown that stimulation of the mPFC terminals in both the mediobasal hypothalamus (MBH) and lateral hypothalamus (LH) regions elicited violent aggression (Biro et al., 2018), and PV^+^ interneurons exclusively contact nearly every local pyramidal neuron to target the soma and proximal dendrites, which enables them to synchronize networks and transfer inhibitory regulation (Packer and Yuste, 2011). Therefore, we hypothesize that PV^+^ interneurons in this region suppress aggressive behaviors by inhibiting pyramidal neurons that modulate aggression by projecting to downstream brain regions.

The other is by the integration of information input to the IL; these abnormal behavioral manifestations may be caused by problems in the integration of upstream information due to impairment of IL function. In the above investigation of diseases that exhibit an increased aggression phenotype, we found it noteworthy that in human psychiatric disorders, particularly borderline personality disorder, patients exhibit markedly impaired amygdala-PFC connectivity (Kolla et al., 2020). Functional magnetic resonance imaging (fMRI) revealed significantly decreased functional connectivity between the amygdala and mPFC in violent offenders and increased connectivity in non-offender controls (Siep et al., 2019). Children with aggressive behavior also showed deficits in amygdala-mPFC connectivity (Ibrahim et al., 2022). Moreover, morphological research has shown GABAergic projections from the amygdala to the IL (Seo et al., 2016), providing anatomical evidence for our hypothesis.

Such evidence supports our findings that PV^+^ interneurons in the IL subregion of the mPFC, which are primarily responsible for receiving projections from the amygdala in the limbic region, become functionally impaired after exposure to adolescent social isolation, resulting in an inability of the PFC to receive normal afferent information and consequently leading to impaired function.

The combination of these two patterns together may constitutes the modulatory patterns of PV^+^ interneurons on aggression. In the long run, a better understanding of the neural mechanisms underlying social isolation-induced aggression in humans might contribute to the development of intervention methods targeted at preventing inappropriate violence.

Additional electrophysiological recordings should be performed in the future to explore the electrophysiological responses of this population of pyramidal neurons that is impaired after adolescent social isolation and the intrinsic property changes in PV^+^ interneurons that remain detectable. Moreover, further electrophysiological experiments could better define cellular electrophysiological changes (E/I balance) in brain slices before and after CNO administration to reflect the cell activity, changes in the E/I balance of the IL at the microcircuit level, and the loss of input and output of PV^+^ interneurons in the IL region at the circuit level of mice after exposure to chronic social isolation during adolescence.

## Conclusion

This study demonstrates significantly excessive aggressiveness in adult mice caused by social isolation during adolescence but not during adulthood. At the morphological level, such chronic stress during adolescence leads to reduced pyramidal neuron complexity in the mPFC region, together with impaired function of PV^+^ interneurons specific to the IL subregion. Further chemogenetic inhibition or activation of IL-PV^+^ interneurons can mimic or rescue the abnormal behavioral changes caused by adolescent social isolation in adult mice. Overall, our findings demonstrate the negative effects of social isolation in adolescence on individual development and the critical role of IL-PV^+^ interneurons in controlling aggression, enhance the understanding of the neural mechanisms that regulate aggression and provide possible intervention targets for chronic adolescent stress-induced overaggression.

## Data availability statement

The raw data supporting the conclusions of this article will be made available by the authors, without undue reservation.

## Ethics statement

The animal study was reviewed and approved by Institutional Animal Care and Use Committees of Xi’an Jiaotong University (No. XJTULAC2020-1325).

## Author contributions

YL and JY designed the study. XL and HS performed the behavioral experiments and analyzed the data. XL and FW performed the virus and drug injections. XL and HS performed the tissue preparation, immunofluorescent staining, and quantitative analysis of the imaging data. YuZ, XL, and XW performed the Golgi staining. FW, XW, and DC helped to collect the data. DL, YiZ, LZ, and XX interpreted the results and commented on the manuscript. XL, HS, YuZ, and YL wrote and revised the manuscript. YL supervised all aspects of the project. All authors have read and approved the final manuscript

## Abbreviations

AAV2: adeno-associated virus
ACC: anterior cingulate cortex
ANOVA: analysis of variance
BPD: borderline personality disorder
CeA: central nucleus of the amygdala
CNO: clozapine-N-oxide
DREADD: designer receptors exclusively activated by designer drug
E/I: excitatory/inhibitory
fMRI: functional magnetic resonance imaging
GH: group-housed
IL: infralimbic cortex
IPSCs: inhibitory postsynaptic currents
LH: lateral hypothalamus
LS: lateral septum
MBH: mediobasal hypothalamus
mPFC: medial prefrontal cortex
PFC: prefrontal cortex
PrL: prelimbic cortex
PV: parvalbumin
RI: resident-intruder
SAL: saline
SI: social isolation
SST: somatostatin
VIP: vasoactive intestinal peptide.
