# Supplementary material for: Dysregulation of prefrontal parvalbumin interneurons leads to adult aggression induced by social isolation stress during adolescence
Source: Front Mol Neurosci. 2022 Oct 4;15:1010152. doi: 10.3389/fnmol.2022.1010152 (PMC9577330; doi:10.3389/fnmol.2022.1010152)
Supplement: Supplementary file 1 [file Data_Sheet_1.docx]

**
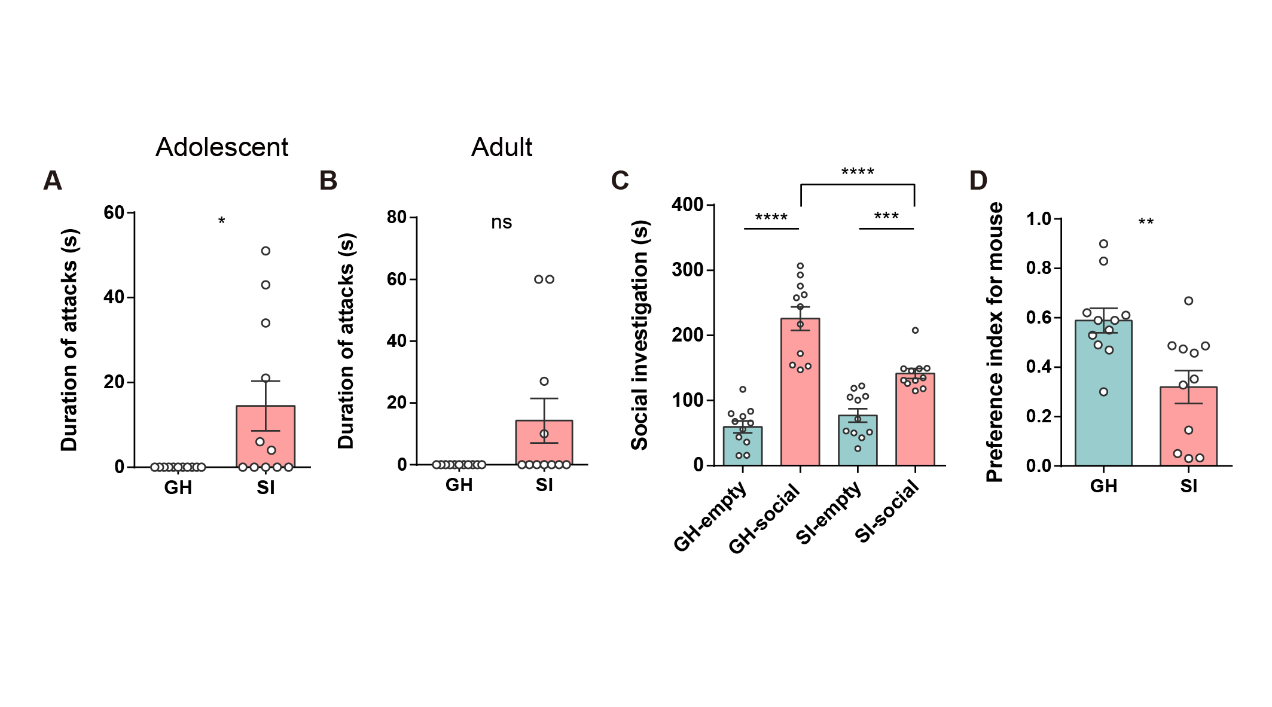
**

**Fig. S1Social isolation during adolescence causes hyperaggression, weight loss, and decreased social preference.(A).**In the adolescent group, SI mice showed an increased attack duration in the free interaction test compared with GH mice (n = 11 mice/group; GH, 0.0±0.0, SI, 14.45±5.878, two-tailed unpaired t test: *P*< 0.05). **(B)**. No obvious alteration in aggression was found in SI mice exposed to adult isolation (n = 11 mice/group; GH, 0.0±0.0, SI, 14.27±7.254, two-tailed unpaired t test: *P* = 0.06). **(C)**. Both GH and SI mice showed a clear preference for the social cage relative to the empty cage, but SI mice showed a distinct decrease in time spent in social investigation compared to GH mice (n = 11 mice/group; GH-empty, 59.66±9.20; GH-social, 225.8±18.06; SI-empty, 77.18±10.34; SI-social, 141.5±7.60, *****P*< 0.0001, ****P*< 0.001 determined by two-way ANOVA with Newman‒Keuls multiple comparisons test). **(D)**. Adolescent SI mice exhibited impaired social preference (n = 11 mice/group; GH, 0.59±0.05, SI, 0.32±0.07, two-tailed unpaired t test: *P*< 0.01). All data are presented as the mean ± SEM.


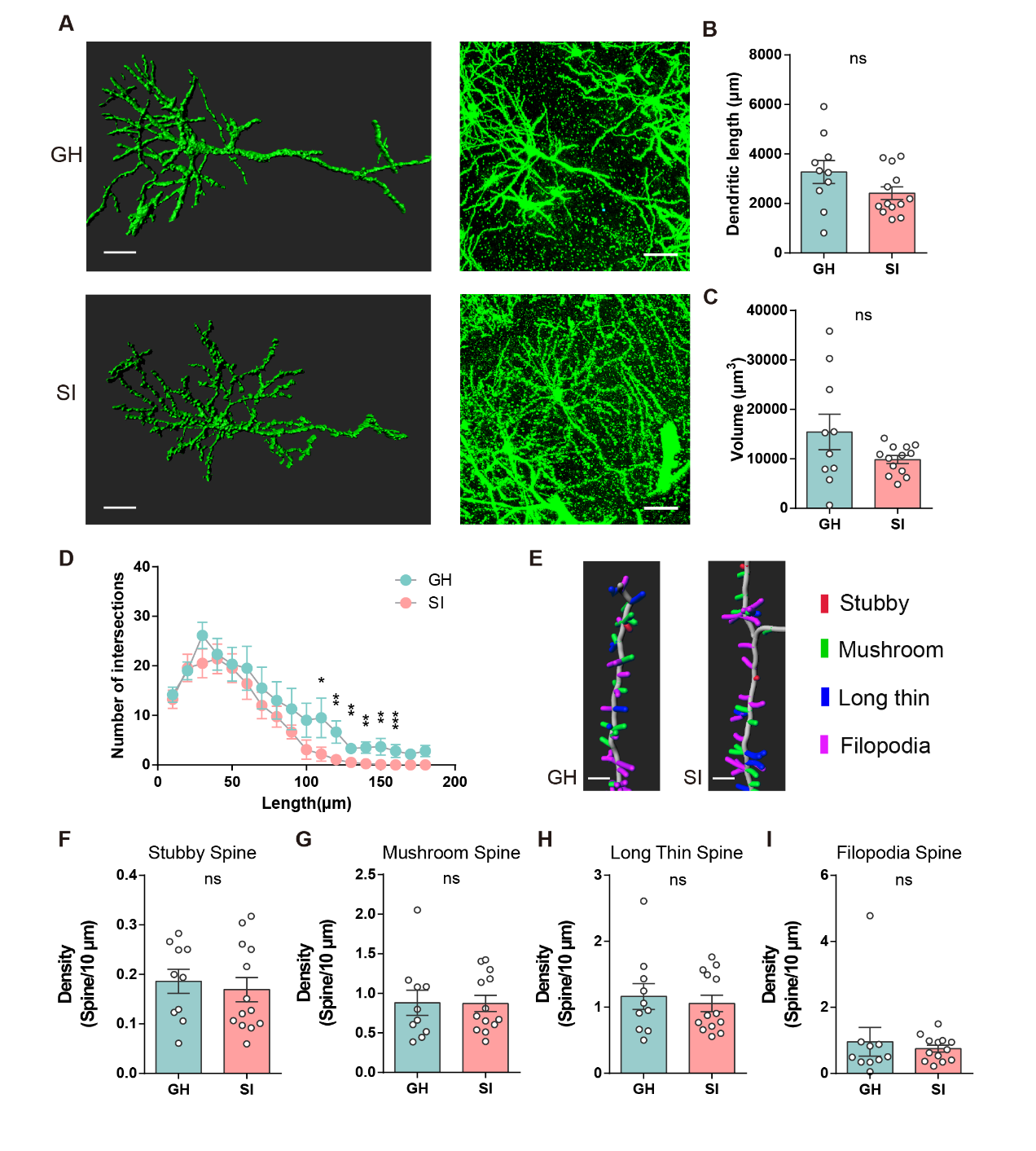


**Fig. S2Impacts of adult social isolation on the structure of dendrites and spines of pyramidal neurons in the mPFC.(A).**Typical confocal diagram showing reconstructed (left) and raw (right) images of pyramidal neurons in the mPFC obtained from GH or SI mouse brains. Scale bars, 30 μm, left; 50 μm, right.For the adult-GH group, 3 neurons in layer 2/3 and 7 neurons in layer 5 from 3 mice were used; in the adult-SI group, 5 neurons in layer 2/3 and 8 neurons in layer 5 from 3 mice were selected.**(B-C)**. Summary of dendritic length **(B)** and volume **(C)** of mPFC pyramidal neurons obtained from the experimental mice (GH, n = 10; SI, n = 13, dendritic length, GH, 3272±464.4, SI, 2416±253.8 *P* = 0.10, volume, GH, 15432±3577, SI, 9866±803.2 *P* = 0.10 determined by two-tailed unpaired t test). **(D)**. Group-housed mice and mice exposed to social isolation during adulthood exhibit comparable levels of complexity of pyramidal neurons in the mPFC. (GH, n = 6; SI, n = 12; **P*< 0.05, ***P*< 0.01 and ****P*< 0.001 determined by two-tailed unpaired t test or the Mann‒Whitney test). **(E)**. Representative Golgi staining images of spines on the dendrites of mPFC pyramidal neurons obtained from adult GH and SI mice. Scale bars, 5 μm. **(F-I)**. Statistical graph of the density of stubby- **(F)**, mushroom- **(G)**, long thin- **(H)**, and filopodia- **(I)** shaped spines on the dendrites of pyramidal neurons in the mPFC from the GH and adult SI-treated mice. (GH, n = 10, SI, n = 13;two-tailed unpaired t test: stubby spine, GH, 0.19±0.02, SI, 0.17±0.02, *P* = 0.64; mushroom spine, GH, 0.88±0.16, SI, 0.87±0.10, *P* = 0.97,two-tailed Mann‒Whitney test: long thin spine, GH, 1.16±0.20, SI, 1.06±0.12,*P* = 0.78; filopodia spine, GH, 0.96±0.43, SI, 0.75±0.11, *P* = 0.40).Data are presented as the mean ± SEM.


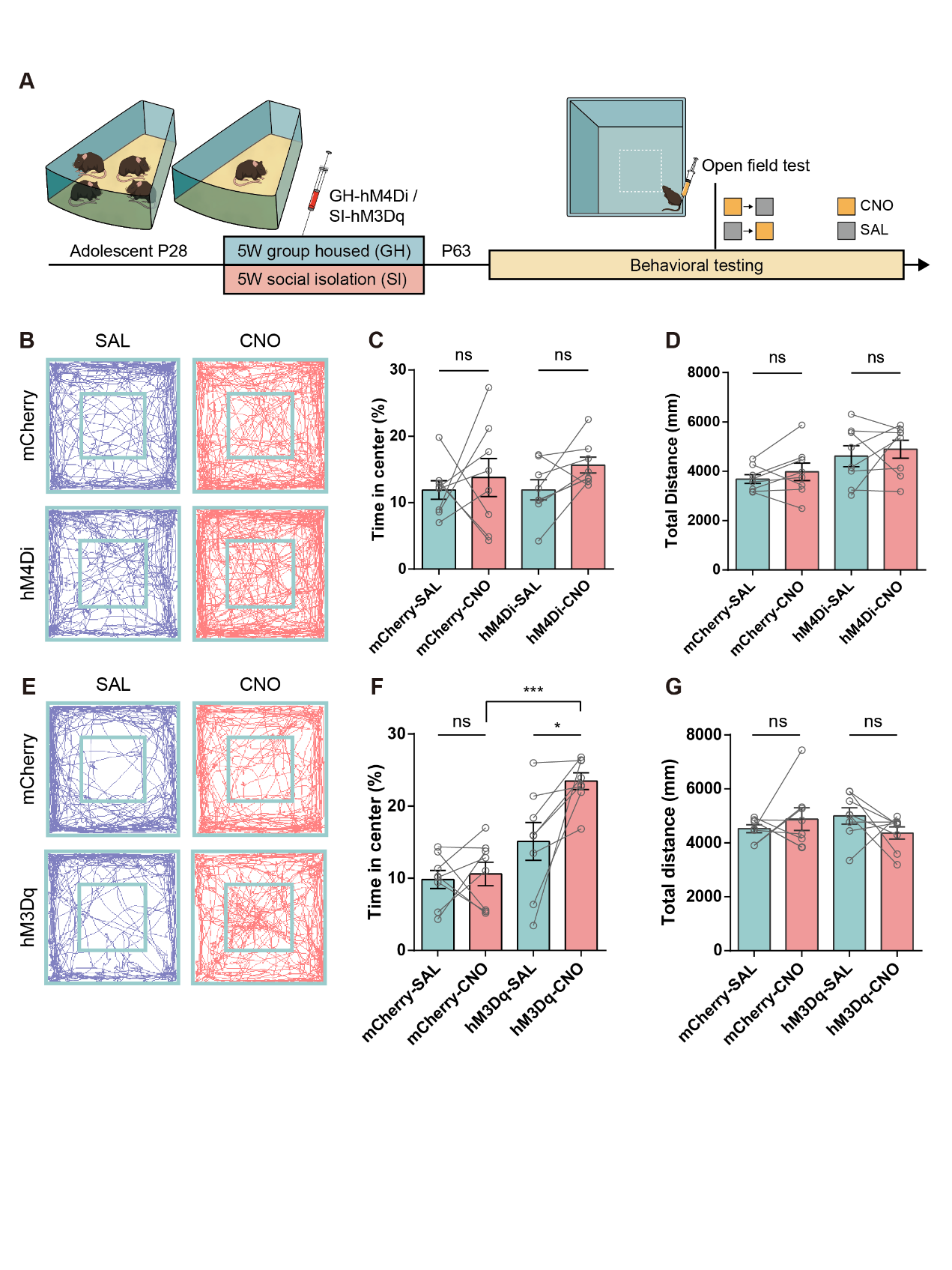


**Fig. S3Chemogenetic stimulation of IL-PV^+^ interneurons increases the time spent in the central area of the open field in adolescent SI mice.(A)**. Timeline showing injection of hM4Di or mCherry into group-housed PV-Cre mice and hM3Dq or mCherry into single-housed PV-Cre mice targeting IL-PV^+^ interneurons. The self-controlled open field test was performed 3 weeks later. Every mouse was tested under both CNO (yellow square) and SAL (gray square) conditions, and each behavioral test was counterbalanced by the order of administration. **(B)**. Arepresentativetrajectory diagram showing the routes of mice in the inhibitory group in the open field. **(C-D)**. Behavioral results from the open-field test showed no obvious change in anxiety **(C)** (n = 8 mice/group, two-tailed paired t test: mCherry, SAL, 11.91±1.40, CNO, 13.80±2.87, *P* = 0.63; hM4Di, SAL, 11.95±1.51, CNO, 15.67±1.19, *P* = 0.06) or locomotion **(D)** (n = 8 mice/group, two-tailed paired t test: mCherry, SAL, 3685±177.0, CNO, 3980±359.9, *P* = 0.31; hM4Di, SAL, 4614±421.8, CNO, 4894±363.9, *P* = 0.56) after CNO administration compared to SAL in both mCherry- and hM4Di-expressing mice. **(E).** Representativetrajectory diagram showing the routes of mice in the activation group in the open field. **(F-G)**. The diagram shows the increased time in the center zone of hM3Dq- but not mCherry-expressing mice treated with CNO compared with SAL administration **(F)** (n = 8 mice/group,two-tailed paired t test: mCherry group, SAL, 9.84±1.26, CNO, 10.61±1.64,*P* = 0.73; hM3Dq group, SAL, 15.11±2.62, CNO, 23.48±1.16, *P*< 0.05). No significant change in motor ability was found in the two groups of mice **(G)** (n = 8 mice/group, mCherry group, SAL, 4524±145.9, CNO, 4878±420.2, *P* = 0.74 followed by thetwo-tailed Wilcoxon matched-pairs signed rank test; hM3Dq group, SAL, 4996±299.9, CNO, 4366±226.1,*P* = 0.20 followed by atwo-tailed paired t test). All data are presented as the mean ± SEM.
